# Developments and perspectives in high-throughput protein glycomics: enabling the analysis of thousands of samples

**DOI:** 10.1093/glycob/cwac026

**Published:** 2022-04-22

**Authors:** Noortje de Haan, Maja Pučić-Baković, Mislav Novokmet, David Falck, Guinevere Lageveen-Kammeijer, Genadij Razdorov, Frano Vučković, Irena Trbojević-Akmačić, Olga Gornik, Maja Hanić, Manfred Wuhrer, Gordan Lauc, the The Human Glycome Project, Andras Guttman, Richard Cummings, Samia Mora, Yoann Rombouts, Andad Mehta

**Affiliations:** Copenhagen Center for Glycomics, University of Copenhagen, Blegdamsvej 3 Copenhagen 2200, Denmark; Genos, Glycoscience Research Laboratory, Borongajska cesta 83h, Zagreb 10000, Croatia; Genos, Glycoscience Research Laboratory, Borongajska cesta 83h, Zagreb 10000, Croatia; Center for Proteomics and Metabolomics, Leiden University Medical Center, Albinusdreef 2, Leiden 2333ZA, The Netherlands; Center for Proteomics and Metabolomics, Leiden University Medical Center, Albinusdreef 2, Leiden 2333ZA, The Netherlands; Genos, Glycoscience Research Laboratory, Borongajska cesta 83h, Zagreb 10000, Croatia; Genos, Glycoscience Research Laboratory, Borongajska cesta 83h, Zagreb 10000, Croatia; Genos, Glycoscience Research Laboratory, Borongajska cesta 83h, Zagreb 10000, Croatia; Faculty of Pharmacy and Biochemistry, University of Zagreb, A. Kovacica 1, Zagreb 10000, Croatia; Genos, Glycoscience Research Laboratory, Borongajska cesta 83h, Zagreb 10000, Croatia; Center for Proteomics and Metabolomics, Leiden University Medical Center, Albinusdreef 2, Leiden 2333ZA, The Netherlands; Genos, Glycoscience Research Laboratory, Borongajska cesta 83h, Zagreb 10000, Croatia; Faculty of Pharmacy and Biochemistry, University of Zagreb, A. Kovacica 1, Zagreb 10000, Croatia

**Keywords:** glycomics, glycoproteomics, high-throughput, mass spectrometry, population studies

## Abstract

Glycans expand the structural complexity of proteins by several orders of magnitude, resulting in a tremendous analytical challenge when including them in biomedical research. Recent glycobiological research is painting a picture in which glycans represent a crucial structural and functional component of the majority of proteins, with alternative glycosylation of proteins and lipids being an important regulatory mechanism in many biological and pathological processes. Since interindividual differences in glycosylation are extensive, large studies are needed to map the structures and to understand the role of glycosylation in human (patho)physiology. Driven by these challenges, methods have emerged, which can tackle the complexity of glycosylation in thousands of samples, also known as high-throughput (HT) glycomics. For facile dissemination and implementation of HT glycomics technology, the sample preparation, analysis, as well as data mining, need to be stable over a long period of time (months/years), amenable to automation, and available to non-specialized laboratories. Current HT glycomics methods mainly focus on protein N-glycosylation and allow to extensively characterize this subset of the human glycome in large numbers of various biological samples. The ultimate goal in HT glycomics is to gain better knowledge and understanding of the complete human glycome using methods that are easy to adapt and implement in (basic) biomedical research. Aiming to promote wider use and development of HT glycomics, here, we present currently available, emerging, and prospective methods and some of their applications, revealing a largely unexplored molecular layer of the complexity of life.

## Introduction

The surfaces of all cells are covered with a dense layer of glycans and nearly all proteins which evolved after the appearance of multicellular life are glycoproteins. This indicates the functional importance of glycans in many biological processes (Walt et al. 2012). Additionally, glycans are important for the success of biopharmaceuticals as they have a large impact on their efficacy and safety (Wang et al. 2019). Glycans have a very large structural diversity, and analyzing interindividual differences in glycosylation, in the form of ABO blood groups, is one of the first examples of successful implementation of a biomarker for personalized medicine (Stanley and Cummings 2015). However, there are many more types of glycosylation and due to their chemical complexity, their non-template-based biosynthesis, and current technological limitations, the field of glycomics still has to develop the capabilities to analyze millions of people as done in genomics and proteomics (Sherman and Salzberg 2020).

The polypeptide part of a protein is largely defined by a genetic template. The analysis of gene sequence variation and transcript levels are a good proxy to study variations in the proteome, but this is not the case with glycans. While glycosylation is considerably heritable (Knezevic et al. 2009), it is inherited as a set of complex traits encoded in large genetic networks (Klaric et al. 2020). This means that genome and transcriptome analyses currently provide limited information about the glycome. Examples of factors influencing glycan biosynthesis are the availability and localization of glycosyltransferases, glycosidases, and nucleotide sugar transporters (Joshi et al. 2018). Thus, to get an accurate view of the glycome, which we, here, define as the complete collection of glycans and glycoconjugates of an organism, biofluid, cell type, or cell population, analytical technologies are required which directly target glycoconjugates isolated from limited amount of a biological sample. While glycomics technology is currently in place to study the glycome of biological systems at a reasonable depth (Ruhaak et al. 2018), such studies are not scalable, and performing HT glycomic analyses on hundreds or even thousands of samples comes at the cost of limited analytical depth. Yet, the ability to reliably analyze the glycome of thousands of samples in a reasonable timeframe and for an acceptable cost is a prerequisite for more widespread glycan analysis in different (basic) biological-, biomedical-, and biomarker-focused population studies. The first study analyzing antibody glycomic signatures of rheumatoid arthritis was performed over 30 years ago and laid the foundation for high-throughput (HT) glycomics and glycomic biomarker discovery (Parekh et al. 1985). The first large-scale study of the glycome, targeting human plasma protein N-glycosylation, was performed over 10 years ago (Knezevic et al. 2009). In the meantime, hundreds of studies have been published, with some of them including thousands of samples (Clerc et al. 2018; Simurina et al. 2018) and revealing the power of glycan-based disease stratification, e.g. in diabetes (Lauc et al. 2010; Juszczak et al. 2019). Several method comparison studies were also performed (Huffman et al. 2014; Reiding et al. 2019), and the results suggest that parts of the HT glycomics field have advanced considerably, showing e.g. high precision, repeatability, and throughput for the analysis of *N*-glycans. In this perspectives paper, we describe glycan analysis methods that have been used in actual HT glycomics studies, that is, comprising thousands of samples and implementing automatable sample preparation and data analysis, and we discuss their advantages, limitations, and perspectives. As current HT glycomics methods mainly focus on protein N-glycosylation, this type of glycosylation is central for the current work. Additionally, the promises and challenges for the HT analysis of other types of glycoconjugates are highlighted.

## Current analytical methods for HT glycomics

While many classes of glycoconjugates exist, including glycoproteins, free oligosaccharides, proteoglycans, glycosaminoglycans, and glycosphingolipids (Varki and Kornfeld 2015), only glycoproteins are currently targeted by HT methods. Within the glycoproteins, again different types of glycosylation can be recognized, which demands a variety of analytical technologies depending on the sample type and research question.

There are roughly three levels on which protein glycosylation can be assessed (Table I). The first, and most widely applied, is the analysis of released glycans. This involves the chemical or enzymatic cleavage of glycans from their protein carrier, which is often followed by chemical labeling prior to detection. Released glycan analysis is a rather generic approach and largely independent of the glycoprotein source (Ruhaak et al. 2018). In addition, it allows the most in-depth structural characterization of glycan species. This is important in glycosylation research, as glycan-epitope variation is subtle but large and has important biological implications. The HT analysis of released *N*-glycans—oligosaccharides attached to the Asn in an Asn-Xxx-Ser/Thr (Xxx ≠ Pro) motif—has matured significantly over the last decade, relying mostly on enzymes that allow the straightforward, largely unbiased, and non-destructive release of *N*-glycans from proteins. These enzymes are available for different classes of *N*-glycans, with PNGase F being most broadly used for human N-glycosylation and with e.g. PNGase A enabling cleavage of plant and insect *N*-glycans. Unfortunately, at the moment, no such tools are available for the other types of protein glycosylation.

**Table I:** Availability and properties of HT glycomics approaches.

Analyte	Pro	Con	Method	Speed	Precision	Structural resolution	Tools for structure and composition assignment	Ref.
Glycan	• Generic approach • Methods available for absolute quantification • High sensitivity • Highest precision and robustness • High level of isomer differentiation	• Sample purity is essential • Information on site- and protein- specificity lost • HT only for *N*-glycans	HILIC– UHPLC–FLD	• 1 sample/ 30 min • 96 samples/ 48 h	Average CV of the 10 most abundant peaks over 2 96-well plates: 1.6%	• Differentiation of various constitutional isomers • Sialic acid linkage differentiation for diantennary glycans	• Retention time • (Tandem) MS • Exoglycosidases	(Huffman et al. 2014; Adamczyk et al. 2017; Reiding et al. 2019)
			CGE–LIF	• 1 sample/ 45 min • 96 samples/ 3 h with 24-capillary CGE	Average CV of the 10 most abundant peaks over 2 96-well plates: 6.9%		• Migration position • Exoglycosidases	(Callewaert et al. 2004; Ruhaak et al. 2010; Huffman et al. 2014; Reiding et al. 2019)
			MALDI–MS	• 1 sample/ 30 s • 96 samples/ 48 min	Average CV of the 10 most abundant peaks over 2 96-well plates: 11.5%	• Sialic acid linkage differentiation for all *N*-glycans	• (Tandem) MS	(Reiding et al. 2014, 2019; Vreeker et al. 2018)
Glycopeptides (e.g. IgG and IgA glycosylation)	• Protein- and site-specific (no pure proteins required) • High sensitivity • *N-* and *O*-glycans	• Optimization of sample preparation often needed • Limited isomer differentiation	LC–MS	• 1 sample/ 13 min • 96 samples/ 24 h	Average CV over all IgG1 glycoforms in the analysis of 20 96-well plates: 8.2%	• No isomer differentiation • Compositional assignment	• (Tandem) MS	(Huffman et al. 2014; Momcilovic et al. 2020)
Intact glycoproteins (e.g. ApoCIII)	• Protein-specific • Often minimal sample preparation • *N-* and *O*-glycans • Complete proteoform assessment	• Rather low sensitivity • No isomer differentiation • Low complexity sample needed • No HT applications yet	MALDI–MS	• 1 sample/ min • 96 samples/ 1.6 h	Average CV over all peaks in the analysis of 1 96-well plates: 15%	• No isomer differentiation • Compositional assignment	• (Tandem) MS	(Nicolardi et al. 2013; Demus et al. 2021)

The second approach involves analysis of glycopeptides obtained after proteolytic cleavage of the glycoproteins of interest. While glycopeptide analysis may in principle be tailored to a broad range of glycoproteins, current HT glycosylation profiling at the glycopeptide level is primarily utilized for the analysis of immunoglobulin N-glycosylation. Examples of HT glycomics for *O*-GalNAc-type (oligosaccharides attached to Ser or Thr residues, initiated by an *N*-acetylgalactosamine) glycopeptides are also reported, such as those covering the O-glycosylated hinge region of human immunoglobulin A1 (IgA1) (Momcilovic et al. 2020; Dotz et al. 2021). Glycopeptide analysis conserves protein- and site-specificity even when the analysis is performed on impure samples. However, the increased number and complexity of the analytes, as compared to released glycans, demand analytical techniques with high sensitivity and resolution. Using a HT glycopeptide-centered approach, mainly monosaccharide composition data are obtained, while structural features are usually not discriminated.

Finally, intact glycoprotein analysis is emerging in the field of HT glycomics, which is so far only sparsely applied on larger sample sets. A notable example is the analysis of intact apolipoprotein CIII that harbors a single O-glycosylation site (Nicolardi et al. 2013; Demus et al. 2021). In principle, intact glycoprotein analysis allows the characterization of the proteoform distribution of an isolated protein but faces limitations regarding sensitivity and glycoform resolution. This is certainly the case for proteins with multiple glycosylation sites. As only the HT analysis of released *N*-glycans as well as glycopeptides of isolated proteins have truly matured, these approaches, together with their indispensable data processing solutions, will be detailed in the following sections.

### Current analytical methods for HT released glycan analysis

**Fig. 1:**
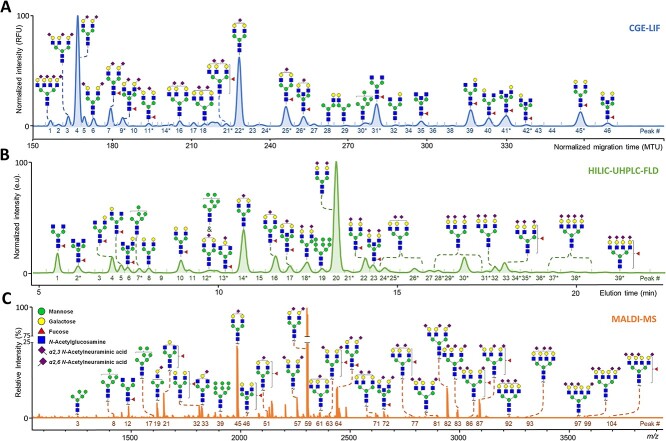
Total serum N-glycosylation profiles as obtained by HILIC–UHPLC–FLD, CGE–LIF, and MALDI–MS. A) Electropherogram by CGE–LIF after APTS labeling (Reiding et al. 2019). B) Chromatogram by HILIC–UHPLC–FLD after 2-AB labeling (Reiding et al. 2019; Zaytseva et al. 2020). C) Mass spectrum by MALDI–FT–ICR–MS after differential sialic acid esterification (Vreeker et al. 2018; Reiding et al. 2019). The *m/z* values of the assigned signals in C) correspond to [M + Na]^+^. HILIC–UHPLC–FLD and CGE–LIF can distinguish differences in branching (galactose arm, bisection, and fucose position). Structures are assigned based on exoglycosidase treatment and/or tandem MS data as well as literature knowledge on *N*-glycan biosynthesis. Some signals correspond to multiple glycan compositions for which the major one is assigned in the figure (CGE–LIF and HILIC–UHPLC–FLD). ^*^For full assignments of each signal detected, see Supplemental Tables SI–SIII.

State-of-the-art HT glycomics approaches for the analysis of released *N*-glycans include hydrophilic-interaction liquid chromatography (HILIC) with fluorescence detection (FLD), capillary gel electrophoresis (CGE) with laser-induced fluorescence (LIF), and matrix-assisted laser desorption/ionization (MALDI)-mass spectrometry (MS) (Fig. 1 and Table I). From Fig. 1, it becomes apparent that the three HT N-glycomics approaches differ in coverage of glycans and resolution of isomers, with the MALDI–MS method providing greater coverage of high mass, highly sialylated, and high antennarity *N*-glycans, while the fluorescence-based methods have advantages in e.g. resolving diantennary glycan isomers differing in arm occupancy (galactosylation of the 6-arm vs. the 3-arm) (Reiding et al. 2019). Although these approaches have been most widely used for *N*-glycan analysis of human plasma proteins, in recent years, they have been readily applied to other types of samples as well.

HILIC–FLD (using high-performance liquid chromatography [HPLC], and more recently, ultrahigh-performance liquid chromatography [UHPLC] systems) allows glycans to be separated based on their size, structure (monosaccharide composition and regioisomerism), and charge. Prior to HILIC–FLD analysis, released glycans are subjected to unbiased and uniform reducing-end labeling to introduce a fluorophore allowing FLD detection (Keser et al. 2018). Reducing-end labeling can be performed via different types of chemistries (Smith et al. 2017) of which reductive amination using 2-aminobenzamide (2-AB) in a one-pot reaction, followed by HILIC solid-phase extraction or porous graphitized carbon (PGC) clean-up (Pralow et al. 2021), is often used in HT glycomics studies (Reiding et al. 2019). This procedure allows sample preparation in a 96-well plate format and high intermediate precision in the separation and quantification of *N*-glycans (Huffman et al. 2014; Reiding et al. 2019). Accordingly, due to its ease and robustness, HILIC–UHPLC of fluorescently labeled glycans regularly serves as a reference method for glycosylation analysis in the biopharmaceutical industry, see e.g. Reusch et al. (2015). Glycan identification is often based on retention time, exoglycosidase treatment, the use of external standards, and existing databases (e.g. GlycoStore, www.glycostore.org, Zhao et al. 2018). Additionally, for a select subgroup of samples in HT glycomics studies, HILIC can be coupled online to MS via electrospray ionization (ESI), making HILIC–UHPLC suitable for the characterization of glycan structures in unknown samples via (tandem) MS (Keser et al. 2018). The latter reduces the speed of analysis and data interpretation but rather can be seen as a synergetic approach to annotate glycan structures in (mixtures of) samples representative of the larger sample set.

CGE–LIF offers an alternative separation strategy for the analysis of released glycans based on differences in their charge-to-size ratio. Similar to HILIC–FLD, this approach allows the efficient separation of neutral and charged glycans in a single run and the partial separation of structural glycan isomers (Ruhaak et al. 2010). The fluorescent reducing-end tag regularly employed in combination with CGE–LIF is 1-aminopyrene-3,6,8-trisulfonic acid (APTS), which can be coupled to the glycans via reductive amination (Ruhaak et al. 2010). In contrast to HILIC–FLD, the gels and buffers used in CGE–LIF analysis are hardly compatible with MS coupling. Therefore, glycan identification is restricted to migration behavior using reference glucose ladders and exoglycosidase treatment in combination with databases. Importantly, CGE–LIF can be multiplexed up to 24-fold on capillary DNA sequencers (Callewaert et al. 2004; Ruhaak et al. 2010), resulting in HT and great perspectives for clinical implementation (Callewaert et al. 2004).

Finally, MALDI–MS analysis (either in combination with broadly available time-of-flight [TOF] analyzers, Reiding et al. 2014, or with ultrahigh-resolution Fourier-transform ion cyclotron resonance [FT-ICR] analyzers, Vreeker et al. 2018) is a valuable tool in HT glycomics (Clerc et al. 2018). Previously, the instability of sialic acids during ionization, causing biases in the detection and quantification of sialylated species, limited the MS analysis of released glycans. This has been addressed by permethylation that stabilizes sialic acids and allows the sensitive MS analysis of complex glycan mixtures. Recent advances in 96-well plate sample preparation have provided important steps toward the HT analysis of permethylated glycans (Shubhakar et al. 2016; Shajahan et al. 2019), resulting in the analysis of serum or plasma *N*-glycans, which can readily be performed on hundreds of samples with high precision using MALDI–MS (Dedova et al. 2018). As an alternative to permethylation, to address the issue of sialic acid instability, different derivatization procedures have recently been developed for sialic acid stabilization (de Haan et al. 2020). Protocols that specifically targeted sialic acids in simple, one-pot, and mild reactions were implemented in HT glycomics protocols, allowing an unmatched sample throughput using MALDI–MS (Reiding et al. 2014; Clerc et al. 2018). As the stabilization strategies introduce a mass difference between sialic acid linkage isomers (α2,3- or α2,6-linkages) via differential esterification, sialic acid linkages are readily distinguished without the use of tandem MS (Reiding et al. 2014). Due to the sialic linkage-specific mass tags, a MALDI–MS compositional analysis of sialylated *N*-glycans provides the specific number of α2,3- and α2,6-linked sialic acid residues per *N*-glycan. Of note, MALDI–MS provides the sialic linkage specification for a diverse range of *N*-glycan species, including tri- and tetra-antennary glycans, while HILIC–FLD and CGE–LIF will only allow to discriminate sialic acid linkages for glycans carrying up to two antennae (Reiding et al. 2019). Furthermore, MALDI–MS/MS allows the compositional analysis of unknown glycans in complex samples, which can be applied on a small subset of samples representative for a complete sample set. Regarding robust quantification, MALDI–MS shares limitations with other MS-based approaches in that response factors between glycans may vary depending on ionization, ion transmission, and detection. These limitations can be addressed to a large extent by implementation of standards, particularly stable isotope-labeled, internal standards. This is, however, not state-of-the-art for current HT MALDI–MS methods. Implementation of such standards will expectedly improve robustness and accuracy of MS methods for both relative and absolute glycan quantification (de Haan et al. 2020).

While the HT methods for released *N*-glycans all provide a certain level of glycan structural elucidation, they often tend not to achieve complete separation and annotation of linkage- and region-isomers. For HILIC–FLD and CGE–LIF, glycan structural assignment is based on standards and glycosidase treatment, and comigrating analytes may confound both structural assignment and quantification. In the case of HILIC–FLD, additional online ESI-MS(/MS) detection can support structural assignment. By contrast, online coupling of CGE–LIF is analytically challenging and not routinely achieved (Bunz et al. 2013). For the MS-based method, isomers form a challenge, with MALDI-TOF-MS/MS supporting the compositional assignment and determination of some structural motifs, while information on decoration of specific antennae and definition of glycosidic linkages will generally remain elusive (Rombouts et al. 2016). This gap can be addressed by the further HT development and implementation of current methods that are already available to perform in-depth structural characterization of released glycans, such as C18- and PGC–LC–MS and ion mobility-MS, as discussed below (Jensen et al. 2012; Gray et al. 2016).

### Current analytical methods for HT glycopeptide analysis

The bottom-up approach to glycoproteomics by reversed-phase-LC coupled via ESI to high-resolution MS is powerful for the identification and quantification of protein- and site-specific glycosylation in complex mixtures. HT analysis of large sample sets requires efficient sample preparation and short LC gradients. It has to be emphasized that HT glycopeptide profiling is currently limited to enriched glycoproteins. When starting from complex matrices, such as serum or plasma, the necessary reduction in sample complexity is commonly achieved by the affinity enrichment of the glycoprotein of interest. Prominent examples can be found in the work performed on site- and subclass-specific glycan profiling of immunoglobulin G (IgG), an important player in adaptive immunity (Gudelj et al. 2018). Workflows are established in which IgG is enriched from serum or plasma using Sepharose-coupled Protein G in a filter plate, which is followed by tryptic cleavage and analysis in a nanoLC–MS setup without further purification (Falck et al. 2017). Such workflows allow the preparation of 384 samples per day and the high precision LC–MS measurement of 96 samples every 24 h (Table I) (de Haan et al. 2019).

Recent technological developments yielded the sensitivity to analyze low abundance glycoproteins and to handle minute amounts of biological material. Stable low-flow ESI conditions of 100–10 nL/min greatly enhanced the ionization efficiency of glycopeptides (Juraschek et al. 1999). Furthermore, the implementation of dopant-enriched nitrogen gas at the interface between the LC and the MS resulted in an enhanced desolvation during ESI, where specific dopants favor glycopeptides over conventional peptides (Falck et al. 2017). The maturation of high-resolution MS analyzers, e.g. TOF and Orbitrap analyzers, makes high-end glycopeptide analysis widely accessible (Yang et al. 2017; Ruhaak et al. 2018). The exploratory identification of glycopeptides in unknown samples is largely aided by fragmentation. Stepped energy collision-induced-dissociation (or higher-energy collision dissociation for Orbitrap instruments) is most widely applied (Yang et al. 2017; Ruhaak et al. 2018), providing a broad range of energies, and consequently, a broad range of fragment ions with structural information content on the glycan portion and peptide sequence. Upcoming hybrid technologies combining radical-medicated and collision-induced fragmentation may provide more extensive peptide sequence information (Reiding et al. 2018). By contrast, for well-characterized glycoproteins, the HT quantification of their glycopeptides is often performed in MS mode, relying on retention time, accurate mass, and isotope pattern matching for analyte quality control (Falck et al. 2017).

**Fig. 2:**
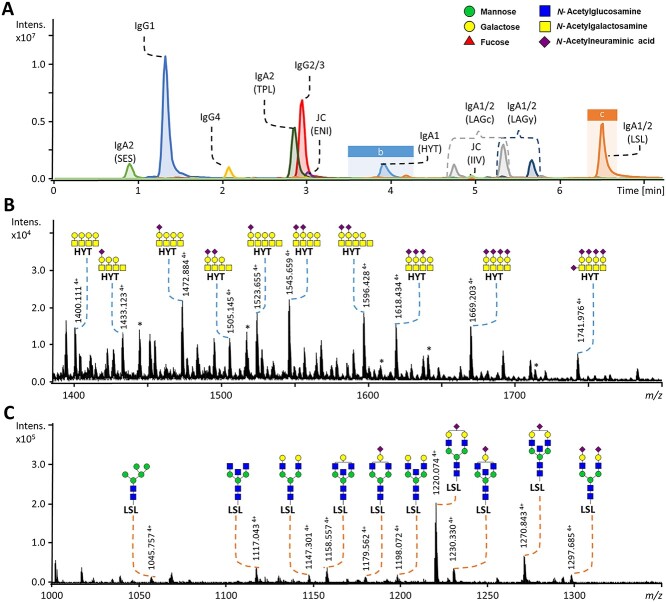
Simultaneous nanoLC-qTOF-MS glycopeptide profiling of IgG and IgA. A) Extracted ion chromatograms for the most abundant glycopeptide per glycosylation site (SES-H4N5F1S1, IgG1-H4N4F1, IgG4-H3N4F1, TPL-H5N5F1S1, IgG2/3-H3N4F1, ENI-H5N4S2, HYT-H4N4S1, LAGc-H5N5F1S2, IIV-H5N5F1S2, LAGy-H5N5F1S2, and LSL-H5N4S1). Protein names and the first three letters of the amino acid sequence of the respective tryptic peptide are given (Momcilovic et al. 2020). Separation was based on the peptide backbones, clustering the analytes with the same peptide sequence but varying glycan portions. The blue and orange boxes indicate the time windows used to generate summed spectra in B) and C), respectively. B) The 10 most abundant glycopeptides from the IgA1 HYT *O*-glycopeptide cluster, with their accurate mass and suggested monosaccharide compositions. C) The 10 most abundant glycopeptides from the IgA1/2 LSL *N*-glycopeptide cluster, with their accurate mass and proposed *N*-glycan structures (based on tandem MS and literature). *Signals not derived from glycopeptides. This figure is adjusted with permission from Momcilovic et al. (2020).

Following the strategy outlined above, LC–MS lends itself to challenging HT investigations, such as the study of antigen-specific antibodies (Larsen et al. 2020) and antibodies from cerebrospinal fluid (Wuhrer et al. 2015). Furthermore, the approach was recently optimized for other proteins, including the different types of immunoglobulins in plasma (Chandler et al. 2019; Momcilovic et al. 2020). To save time and biological material, a recent focus in glycopeptide profiling is the combined purification of multiple proteins of interest, as was successfully shown for the simultaneous analysis of IgG and immunoglobulin A (IgA) from human serum (Fig. 2) (Momcilovic et al. 2020). Soon, HT glycopeptide profiling will likely be attempted for membrane proteins derived from individual cell types and single B-cell clone-derived antibodies (Wojcik et al. 2020). Still, HT glycopeptide analysis is limited to samples of relative low complexity, usually focusing on only one or several enriched glycoproteins. Key in the broader application of HT glycoproteomics are miniaturized sample preparation and advances in data analysis software tools. Additionally, current HT glycoproteomic approaches often only provide limited glycan structural information such as monosaccharide compositional data. More extensive pre-MS separation to resolve isomers and emerging hybrid fragmentation strategies can boost the level of structural information in the future as discussed below (Reiding et al. 2018; Zhu et al. 2020).

### Current analytical methods for HT glycomics data processing and reporting

Similar to other omics, glycomics heavily relies on tailored computational tools for data processing, which are extensively reviewed elsewhere (Lisacek et al. 2017; Mariethoz et al. 2018). As a first step in HT glycomics data processing workflows, analytes of interest need to be identified. For routinely analyzed sample types, looking at either released glycans or glycopeptides, one can rely on matching of prior identifications using (normalized) retention/migration time and, for MS-based approaches, precursor mass. New glycoforms can be explored based on database and literature searches by matching glucose units or *m/z* values. For (LC–)MS data, tools are available, including Glycoworkbench (Damerell et al. 2012) and GlycopeptideGraphMS (Choo et al. 2019), that identify glycoforms based on retention time differences and mass increments compared to known structures. Furthermore, MS/MS data of glycopeptides can be mined using proteomics software tools such as Byonic. For the interpretation of released glycan MS/MS data, spectral databases are available to help in the assignment of negative mode fragmentation spectra (Campbell et al. 2014), but manual interpretation of the spectra may additionally be required, aided by Glycoworkbench (Damerell et al. 2012).

Having established a list of target analytes, relative glycoform quantification can be achieved by HappyTools (Jansen et al. 2018) for LC–FLD (released glycans), glyXtool^CE^ (Hennig et al. 2011) for CGE–LIF (released glycans), MassyTools (Jansen et al. 2015) for direct ESI- or MALDI–MS (released glycans and glycopeptides), LaCyTools (Jansen et al. 2016), and Skyline (MacLean et al. 2010) for LC–MS (released glycans and glycopeptides). An essential feature of all these tools is the automated correction for measurement variations in migration/retention time (alignment) and/or *m/z* values using dataset-specific calibrants, targets, and boundaries. For MS-based approaches, covering many degrees of variability, for example, MS peak shape, adducts, charge states, and isotopologues, is essential for a robust analysis. Curation of individual features can resolve isobaric interferences. All listed tools output data on the quality of individual analytes and measurements/samples, aiding (semi)automatic curation of individual analytes and measurements. In all separation-based approaches, including LC–MS, it is important to cover the complete chromatographic/electrophoretic peaks during quantification, which, in the case of variations in retention or migration times, requires alignment tools and accurate peak detection algorithms. This additional level of variation, next to the *m/z* dimension, makes the processing of glycoproteomics LC–MS data particularly challenging, and improved tools are needed to support curation of these complex datasets.

Most commonly, total area normalization—per protein and site, if available—is used to obtain the final relative quantification of glycoforms. The absolute quantification of released glycans and glycopeptides is still in its infancy and is not implemented in HT approaches. Absolute quantification in LC–FLD and CE–LIF would need consistent, close-to-complete glycan release and labeling. Current methods are not validated for these aspects. Regarding quantitative detection by MS, while isotopic labeled standards and labels are available and have shown applicability (Varadi et al. 2016; Kalmar et al. 2020; Wang et al. 2021), challenges remain in glycoform coverage, input material normalization, and availability and costs of the required reagents. A miniaturized sample preparation and the focus on specific glycosylation features during analysis may help to address some of these issues. The software tools available for HT signal integration and quality control, as described above, allow the implementation of absolute quantification using minor adjustments.

**Fig. 3:**
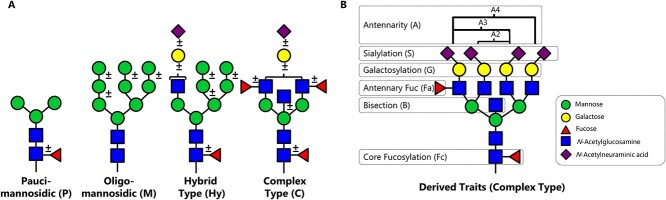
Derived N-glycosylation traits. A) *N*-glycans can be divided into four types, representing their maturation throughout the biosynthetic pathway. B) Per N-glycosylation type, different traits can be calculated as shown here for complex type *N*-glycans (including their common abbreviations). The calculation of derived glycosylation traits allows the representation of basic glycan biosynthesis steps and enhances data precision.

Glycomics data are multivariate and it remains a challenge to give a comprehensive and intelligible overview of complex glycomes. For this purpose, automated visualization tools, such as Glynsight (Alocci et al. 2018), have been developed. Alternative approaches include the reduction of glycoform patterns to derived traits (Fig. 3), which are summed features that often follow basic biosynthetic steps such as galactosylation, sialylation, fucosylation, etc. (Bladergroen et al. 2015). Next to featuring improved precision, derived traits also address a major drawback of total area normalization by removing many interdependencies between analyte quantities as well as reducing the number of variables for statistical evaluation (Bladergroen et al. 2015). A pitfall of this approach is that one can overlook information on specific glycan structures when not defined in the specified traits, especially when not all of these glycan features were resolved by the used analytical strategy.

## Recommendations in study design and quality assurance

HT glycomics studies aim to reveal the association of glycosylation with phenotypes or genotypes. Due to large interindividual differences in human protein glycosylation and the multivariable outcome of typical glycomics analyses, large studies are needed to provide the power to assess the association of glycosylation with human physiology and disease (Ugrina et al. 2017). In a proper study design, the known confounders of glycosylation are equally distributed between the different biological groups of interest. Important confounders to take into account include a diverse range or parameters of the individuals such as age, sex, body mass index (BMI), geographical origin, inflammatory status, pregnancy status, and use of medication (immune suppressors, blood transfusion, [glycosylated] intravenous IgG, monoclonal antibody drugs, etc.) (Knezevic et al. 2010; Ugrina et al. 2017). Additionally, information on sample acquisition centers and sample storage is important, although the latter seems to be less critical for glycomics than for e.g. proteomics studies, as glycans are rather stable over a range of different storage conditions (Dedova et al. 2018; Vreeker et al. 2018). Hemolysis has been found to negatively affect the analyses of the total plasma *N*-glycome (Dedova et al. 2018). While serum and plasma appear to be equally suitable for IgG glycosylation analysis, with no noticeable bias, they slightly differ with respect to the obtained total released glycan profiles, reflecting the differences in glycoprotein composition (Adamczyk et al. 2013; Dedova et al. 2018).

Typical HT glycomics studies comprise hundreds to thousands of samples, and their preparation and measurement can take up to several weeks or months. During that time, many experimental conditions can vary and influence the measurements. In order to minimize or even eliminate the effects of experimental variation, two principles of experimental design should be applied: replication and (blocked) randomization (Ugrina et al. 2017). Replication involves the inclusion of a standard sample throughout the different predefined study batches (e.g. 96-well plates and sample preparation or measurement days), allowing to detect systematic biases between batches as well as other non-systematic biases throughout a study. The replication standard should be representative for the samples analyzed, for example, being a pool of a sample subset. Monitoring batch- or cohort-specific effects may allow for statistical correction thereof, and monitoring for overall repeatability within a dataset provides a measure of data quality. While in-house replication standards are well established in the larger HT glycomics laboratories, global standards to compare glycomics data through space and time are largely lacking (De Leoz et al. 2020).

To control for the effect of experimental factors on glycan measurements, the blocked randomization of samples between batches is critical. In such a design, every batch (block) maintains a constant distribution of known experimental and main biological factors (Leek et al. 2010). To allow for a blocked study design, information about the main confounders is required before analysis.

## Challenges and perspectives in technological developments

Current analytical methodologies allow the HT analysis of released *N*-glycans derived from a wide variety of liquid biopsies and isolated proteins. In addition, HT glycomics successfully targets *N*- and *O*-GalNAc-glycopeptides of a select set of glycoproteins (Table II). As the importance of glycosylation in basic biological processes is increasingly recognized, the demands on technical capabilities of glycoanalytical methods are getting higher. Deep structural characterization of glycans, in combination with their protein carrier, is required in settings where glycan structure–function relationships are investigated. Another methodological challenge lies in determining the spatial distribution of specific glycans and glycoproteins in tissues and cells. The question of glycoconjugate localization goes hand in hand with sensitivity. While single-cell sensitivity is now relatively mainstream in transcriptomics and emerging in proteomics, this is still a long way off in glycomics. Finally, far from trivial is the accessibility of HT glycomics to the non-specialist. Most HT glycomics methods are currently not ready to be broadly implemented as routine research platforms.

**Table II:** Current status of, and perspectives for, HT glycomics methods to dissect the human glycome.

	Current		Emerging	Future
**Sample type**	• Plasma • Serum • Urine	• CSF • Saliva	• Tissue • Cell lines	• Single cells
**Analyte**	• *N*-glycans • Glycopeptides of a select set of isolated proteins in HT mode • Glycopeptides of complex mixtures in low throughput		• *O*-glycans • Glycopeptides of a broader set of isolated proteins • Intact glycoproteins	• Glycopeptides in complex matrices/mixtures^a^ • GAGs • GSL glycans
**Analytical depth**	• *N*-glycan isomer information (limited) • Protein specificity • Site specificity		• More extensive isomer information for all glycan types • Proteoform information (intact)	• Isomer information at the glycopeptide level
**Research field**	• Biopharma • Preclinical • Biomedical • Specialized glycan labs		• Clinical • Non-glycan specialized labs	• Routine clinical diagnostics

^a^Robust quantification in a HT manner of all glycopeptides in e.g. total plasma; CSF, cerebrospinal fluid; GAG, glycosaminoglycan; GSL, glycosphingolipid.

The extent of structural elucidation in current HT methods is, while still limited, most advanced in the analysis of released *N*-glycans. HT structural elucidation is only just emerging for other glycan types and at the level of glycopeptides. Advances in the current techniques are expected while maintaining robustness and throughput (Peng et al. 2021). Ion mobility-MS shows great potential to contribute to the HT separation of glycan (fragment) isomers (Gray et al. 2016). Additionally, liquid-phase separation modules, such as PGC–LC, already provide unmatched structural isomer separation (Jensen et al. 2012; Zhu et al. 2020). These methods need to improve in both robustness and ease of implementation to play a role in HT glycomics. For PGC, desired column formats are often not available, requiring in-house column packing for nano LC and capillary LC (Zhang et al. 2020). Also, sample preparation for PGC is rather laborious even after recent adaptations to the 96-well plate format (Zhang et al. 2020).

Throughput of LC–MS based methods can be extended via sample multiplexing using isobaric tandem mass tags as is now common in proteomics (Afiuni-Zadeh et al. 2016; Jiang et al. 2020). For glycopeptides, similar approaches may now be combined with efficient fragmentation methods targeting both the peptide and glycan portion of the glycoconjugate (Reiding et al. 2018). The implementation of these developments in HT glycomics is highly dependent on the codevelopment, and active maintenance, of software packages that can manage the resulting multidimensional data and that allow the identification, quantification, and quality control of the data in a HT manner. Furthermore, pure, accessible, and well-defined chemoenzymatically generated standards will tremendously help in the annotation of glycan structural features in complex samples.

MS of glycopeptides is gaining importance in HT studies as it allows the analysis of glycosylation in a protein- and site-specific ways in conjunction with identifying other posttranslational modifications (PTMs). This strength is, for example, exploited in multiple attribute monitoring in biopharma (Lippold et al. 2020). Fully automated tools integrating identification, parameter optimization, quantification, visualization, and ideally statistical treatment of features will be an important step toward democratizing HT glycomics to the wider scientific community.

MS of intact proteins allows the analysis of glycosylation in a protein-specific way along with other protein structural features and PTMs. A big advantage of intact protein analysis is the often very simple sample preparation workflow, favoring the use of this approach in biopharma for batch release as well as in clinical diagnostics (de Haan, Wuhrer et al. 2020). For intact protein analysis, the resolution and confident identification of the different proteoforms is often challenging, which, together with the limited sensitivity, hampers its use in HT glycomics to date.

Next to the use of glycan-targeting antibodies and specific lectins, protein *N*-glycan spatial distribution in tissues is currently investigated using advanced MS imaging (MSI) workflows. The spatial resolution of these methods is in the range of 5–20 μm and not yet advanced enough to study single cells (Dilillo et al. 2018). However, structural resolution is often higher than by staining with antibodies or lectins, and technological developments in this area are expected to improve spatial resolution in the near future. Developments in analysis speed and especially data processing tools will determine the applicability of MSI in a HT setting.

Protein N-glycosylation and (to a certain extent) *O*-GalNAc-glycosylation have increasingly received attention, while other groups of glycoconjugates, including different types of O-glycosylation, glycosaminoglycans, and glycosphingolipid glycans, remain vastly understudied. These functionally important groups of biomolecules deserve to be explored as they play central roles in many biological processes (Varki and Kornfeld 2015). Hence, HT glycomics method development will need a certain level of diversification for broad glycome coverage (Table II). These developments should in particular focus on simple and miniaturized sample preparation of the different types of analytes.

Also, the type of samples targeted by HT glycomics should be extended in the future. While the current focus is mainly on liquid biopsies, with plasma and serum being most thoroughly explored, glycans are of great importance at the interface between cells or tissues. The optimization of analytical sensitivity, and sample preparation workflows targeting cell surface and tissue extracellular matrix glycomes, will allow the exploration of this mostly uncovered layer of the human glycome with, as holy grail, single-cell glycomics. Important factors in enhancing sensitivity are simplified and miniaturized sample preparation protocols in combination with ultra-low flow separation modules coupled to MS.

## Perspectives for the wider application of HT glycomics

Next to basic biological research, glycomics has found its way in biomarker discovery, personalized health care, as well as drug and vaccine development. The biomarker potential of glycans was identified in numerous studies (Kavur et al. 2021) and the clinical analysis of glycosylation is routinely used for the diagnostics of congenital disorders of glycosylation (Post and Lefeber 2019). Another example of a clinically validated assay that is based on glycosylation profiling is the Glyco Liver Profile test from Helena Biosciences (Vanderschaeghe et al. 2010). Further implementation of glycomics tests in the clinic is limited due to the absence of validated diagnostic instruments and analytical standards. Another key obstacle for more widespread use of glycan biomarkers is the very large interindividual variability of glycosylation which is blurring diagnostically relevant information. As mentioned previously, genetics (Štambuk et al. 2020), age (Štambuk et al. 2020), BMI (Greto et al. 2021), and gonadal hormones (Ercan et al. 2017) are a few of the known factors that significantly contribute to interindividual variation in glycome composition, but there may be others that are still not identified. Furthermore, since glycan biomarkers are dynamic and change through time, contrary to genetic data that need to be generated only once, glycans need to be measured in a longitudinal manner. Although this dynamic nature of glycosylation can be seen as a challenge, it also enables the development of methods for the longitudinal evaluation of effects of different pharmacological and lifestyle interventions aimed at improving health. Initial studies have shown that plasma *N*-glycans can indeed be altered with weight loss (Greto et al. 2021), fecal microbiome transplant (Monaghan et al. 2019), hormone replacement (Jurić et al. 2020), and exercise (Tijardović et al. 2019), which confirms their significant potential as predictive biomarkers for disease prevention.

Glycan analytics is currently indispensable in the biopharmaceutical industry. Since most of the therapeutic monoclonal antibody proteins are glycosylated and glycans significantly affect many aspects of their structure and function, glycoengineering is developing into an integral component of drug development (Mastrangeli et al. 2019). Other opportunities in monoclonal antibody drug development lay in targeting carbohydrate-based antigens. However, knowledge about the regulation of glycosylation is still rudimentary and large glycomics studies will be needed to understand the regulation of glycosylation and its tissue-specific differences (Hakomori 2002; Landini et al. 2021). Finally, while pharmacogenomics is already an established field with multiple clinical applications, pharmacoglycomics is still understudied and its relevance is starting to be acknowledged (Özdemir et al. 2020). Glycosylation is known to affect the drug-binding properties of certain proteins, implying that interindividual differences in glycosylation may affect drug bioavailability with potential in precision medicine (Behan et al. 2013; Hayes et al. 2014).

## Funding

This work was supported by the European Structural and Investment Funds IRI “CardioMetabolic” grant (#KK.01.2.1.02.0321), the Centre of Competence in Molecular Diagnostics grant (#KK.01.2.2.03.0006), the Croatian National Centre of Research Excellence in Personalized Healthcare grant (#KK.01.1.1.01.0010), the Dutch Research Council project “Proteoform-resolved pharmacokinetics of biopharmaceuticals” (ENPPS.LIFT.019.012), and the European Research Council (ERC) project “GlycoSkin” (H2020-ERC; 772735).

*Conflict of interest statement*: Maja Pučić-Baković, Mislav Novokmet, Genadij Razdorov, Frano Vučković, Irena Trbojević-Akmačić, and Maja Hanić are employed at, and Gordan Lauc is the founder of, Genos Ltd, a private research organization that specializes in HT glycomic analysis and has several patents in this field. Manfred Wuhrer is the inventor on a patent on MS glycan profiling: EP 3457123 A4.

## Abbreviations

2-AB: 2-aminobenzamide

APTS: 1-aminopyrene-3,6,8-trisulfonic acid

BMI: body mass index

CGE: capillary gel electrophoresis

ESI: electrospray ionization

FLD: fluorescence detection

FT-ICR: Fourier-transform ion cyclotron resonance

HILIC: hydrophilic-interaction liquid chromatography

HPLC: high-performance liquid chromatography

HT: high-throughput

Ig: immunoglobulin

IgA: immunoglobulin A

IgG: immunoglobulin G

LIF: laser-induced fluorescence

MALDI: matrix-assisted laser desorption/ionization

MS: mass spectrometry

MSI: mass spectrometry imaging

PGC: porous graphitized carbon

TOF: time-of-flight

UHPLC: ultrahigh-performance liquid chromatography

## Supplementary Material

20220412_SupTables_cwac026
